# A theoretical study of chemical bonding and topological and electrostatic properties of the anti-leprosy drug dapsone

**DOI:** 10.1007/s00894-020-04393-6

**Published:** 2020-05-15

**Authors:** Niranjana Devi Rajendran, Natarajan Mookan, Israel Samuel, Sarath Babu Mookan, Govindarajan Munusamy, Selvaraj Gurudeeban, Satyavani Kaliamurthi

**Affiliations:** 1 College of Biological, Chemical Sciences and Engineering, Jiaxing University, Jiaxing, Zhejiang 314001 China; 2 Department of Chemistry, Gargi College, University of Delhi, New Delhi, 110049 India; 3 Research Centre of Physics, Fatima College, Madurai, 625018 Tamil Nadu China; 4 Research and Postgraduate, Department of Physics, The American College, Madurai, 625002 Tamil Nadu India; 5 Arignar Anna Government Arts and Science College, Nehru Nagar, Karaikal, Puducherry State India; 6 Center of Interdisciplinary Science-Computational Life Sciences, College of Food Science and Engineering, Henan University of Technology, Zhengzhou High-tech Industrial Development Zone, 100 Lianhua Street, Zhengzhou, Henan 450001 China

**Keywords:** Hirshfeld surface analysis, Quantum chemical calculations, Charge density analysis, Bond topological properties, Electrostatic properties

## Abstract

The theoretical charge density study for the gas phase of anti-leprosy drug Dapsone has been carried out in the light of the theory of atoms in molecules using density functional theory employing B3LYP(6-311G++(d, p) hybrid functional completed with dispersion corrections. The Hirshfeld surface analysis as well as fingerprint plots has been utilized to visualize and quantify the intermolecular contacts present in the molecule. The topological properties such as electron density and its Laplacian, delocalization index have been elucidated to throw light into the chemical bonding and atomic and molecular details. The electron localization function has been used to visualize and deduce information on the lone pair and the subshells of the Cl atom. The electrostatic potential visualizes the positive and negative electrostatic potential regions which are susceptible to nucleophilic and electrophilic attack. On the whole, this study provides an exact mechanism, interaction, and topological and electrostatic properties of the drug through theoretical insights which all will be a platform for our further investigation of the interaction between dapsone and dihydropteroate synthase (DHPS).

## Introduction

Dapsone, an antibacterial, is primarily and effectively used in the leprosy treatment which is also used in combination with rifampicin and clofazimine for the treatment of *Mycobacterium leprae* infections (leprosy) [1–4], malaria [5–7], and pneumocystis pneumonia (PCP) [8–10]. Specifically, dapsone stops the bacterial dihydrofolic acid synthesis through the process of binding itself in the active site of the enzyme named 6-hydroxymethyl-7,8-dihydropteroate synthase (DHPS), which takes part in the condensation of para-aminobenzoic acid (pABA) with 6-hydroxymethyl-7,8-dihydropterin-pyrophosphate to form 7,8-dihydropteroate and pyrophosphate [11]. Moreover, dapsone competes with para-aminobenzoate on the active site of DHPS and inhibits the bacterial dihydrofolic acid synthesis [12]. In present literature, Mendes et al. carried out the geometric and electronic study of dapsone and discussed the symmetric and asymmetric conformational isomer of the molecule [13]. Borges et al. carried out the first density functional study of the dapsone derivatives on methemoglobin [14]. Bhattacharya et al. revisited dapsone photophysics in a different solvent [15]. To the best of our knowledge, neither the study of theoretical charge density nor the topological properties for the gas phase of the molecule have been discussed in the literature.

The elaborate study on charge density in the light of AIM theory and electrostatic mapping of a molecule is very important due to their crucial application in determining the interaction of pharmaceutical compounds with a biomolecule. As referred to Bader the pioneer stated in AIM theory: “A Quantum theory [16], while the theory has its origin in quantum mechanics, its vehicle of expression, is the charge density”. The charge density being the most important property is much useful in finding one-electron properties, ground-state properties of the molecules, type and the strength of the chemical bonding between the atoms, information on lattice energies, orbital locating and molecular interactions, etc. Especially hydrogen bonding serves as the principal source for the intermolecular interactions which further helps in tailoring more compounds with desired physical and chemical properties.

In the present work, an attempt has been made to reconstruct the charge density mapping of dapsone (4-[(4-aminobenzene) sulfonyl] aniline) molecule using theoretical models and is thoroughly analyzed with the help of Quantum theory of atoms in molecules for the intuitive information on the molecule itself. In a theoretical model such as DFT (density functional theory) [17], a free dapsone molecule has been treated and analyzed for its electronic properties. The Hirshfeld surface analysis [18, 19] has been carried out for a clear understanding of the intermolecular hydrogen bonding interactions, and the fingerprint plot has been mapped for calculating the percentage of contribution of various bonds present in the molecule. The topology of the charge density has been analyzed, and the critical points in the charge density have been determined. The Lipinski rule of five has been calculated in order to appreciate the potential of the drug. Electrostatic potential (ESP) surfaces have been examined for the identification of not only the electrophilic and nucleophilic regions of the molecule but also understanding the lock and key mechanism. The results obtained from this study will be a manifesto for the further exploration of interpretation of the drug-receptor interactions between the dapsone molecule and the enzyme of dihydropteroate synthase (DHPS).

## Computational details

The experimentally determined position values of the atoms of dapsone have been given as input for the theoretical optimization in GAUSSIAN09 software package [20] with DFT method using (B3LYP\6-311G++(d, p)) level of theory [21, 22]. The single-point calculations have been performed once the convergence has been reached. The absence of imaginary frequencies has shown that the minimum energy structure has been achieved. The obtained wave function for the gas phase of the dapsone molecule has been given as an input to the AIM ALL package [23] for calculating the topological properties. The AIM-UC 4.0. [24] and MULTIWFN [25] software packages have been utilized for mapping purposes.

## Results and discussion

### Description of structure

**Fig. 1:**
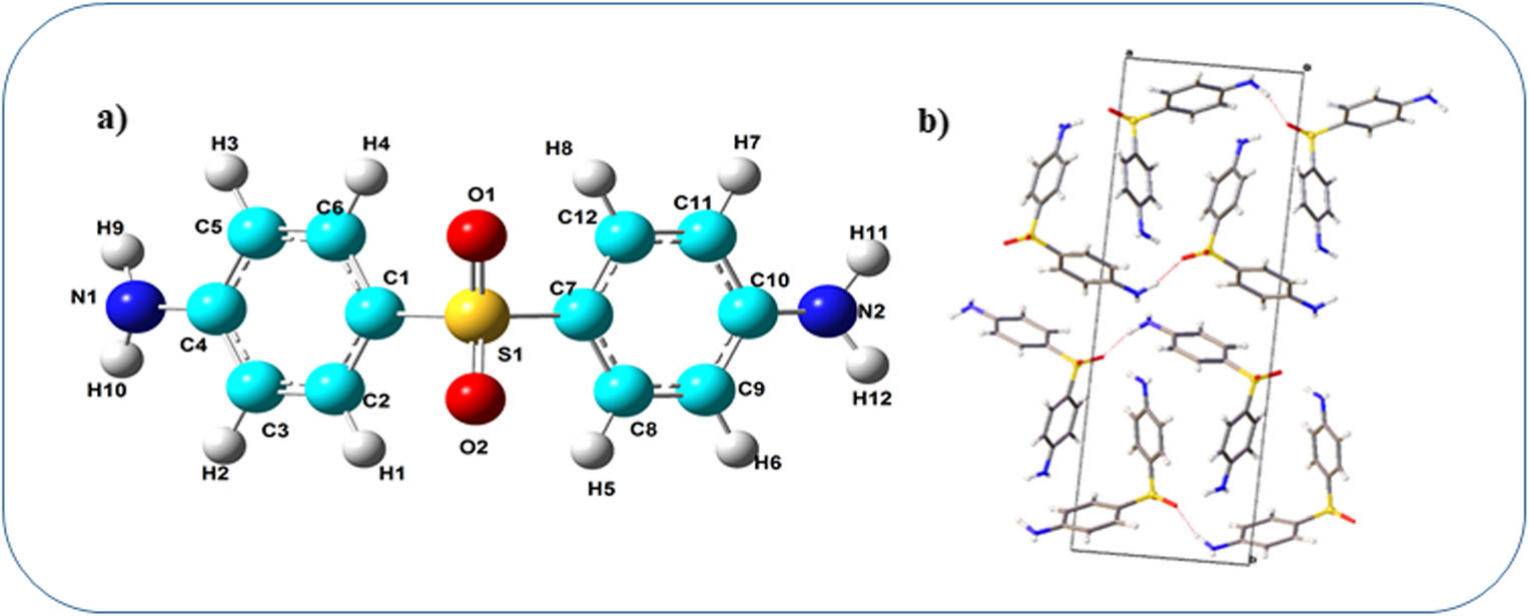
**a** Optimized structure of dapsone. **b** View of the crystal packing in dapsone (along the c* axis)

The minimum energy structure of the dapsone molecule has been shown in Fig. 1a. In both the aniline rings, the standard values for C–C and C–H bonds are 1.39 Å and 1.09 Å, and the values from our theoretical calculations lie in the range 1.387–1.406 Å and 1.083–1.085 Å which agrees well with the standard values [26]. The expected bond length for the C–N bond is 1.48 Å, but the optimized bond lengths for the C–N bond are in the range 1.387–1.388 Å where the decrease in bond length might be due to the resonance among C–N bonds. The calculated angle for the plane O1-S1-O2 is found to be 120° which suggests the trigonal planar geometry and sp2 hybridization state for the corresponding central S1 atom.

The shapes of the two amine groups are found to be a slight tetrahedral with the expected bond angle 113°. The atoms of N1 and N2 make equal dihedral angles with the respective planes C3-C4-C5 and C9-C10-C11 and are found to be 177°.The torsion angles which are formed by the atoms C1 and C7 with O2-S1-O1 plane individually are found to be 123.4° which clearly suggest the symmetry of the molecule.

### Hydrogen bonding interaction

In order to better understand the packing and interactions of the molecule with the neighboring molecules, the information from the experimental cif file has been given as the input for CrystalExplorer software 3.0 [18, 27]. The packing of the molecules in the crystal lattice (Fig. 1b) is viewed along c* axis and the intermolecular interactions are sustained by Van der Waals interactions.

**Fig. 2:**
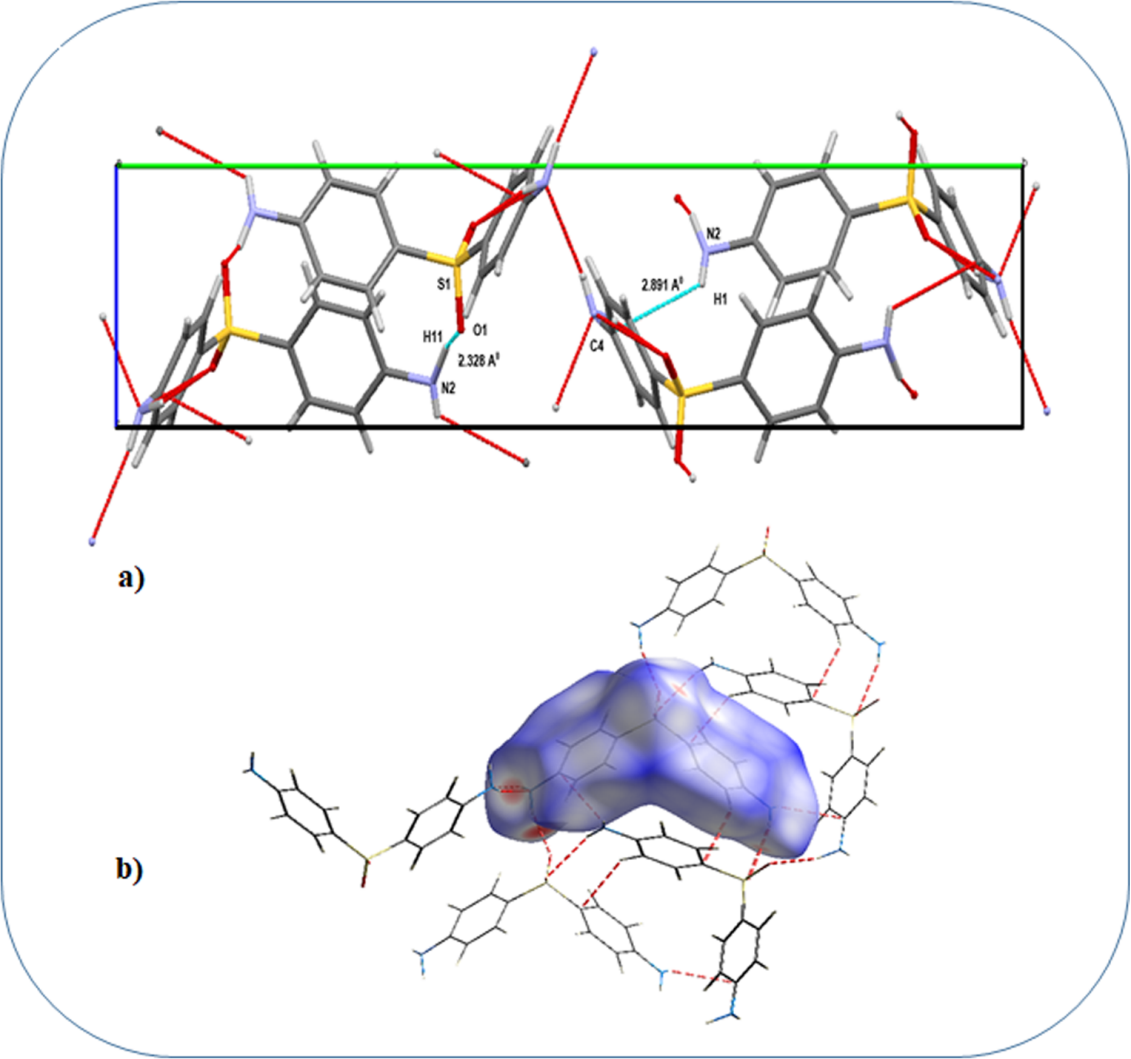
**a** View of the intermolecular hydrogen bonds (along with **a** axis). **b** Visualization of the Hirshfeld surface of the strong interactions

The molecular packing of the crystal is stabilized by N–H…O, N–H…N, and π…π types of intermolecular interactions (Fig. 2) and is classified as strong and weak interactions with respect to their H…A distances. From Fig. 2a, it is obvious that there is no intramolecular N–H…O hydrogen bond formation, but two types of intermolecular N–H…O hydrogen bond formation is possible.

The hydrogen bond parameters of the strongest N1–H9…O2 interaction are: H9…O2; 1.990Å and N1–H9, 1.009Å, and the angle is 159.46°, and another weak N2–H11…O1 interaction are: H11…O1; 2.423Å, N2-H11; 1.009Å, and the angle is 145.29°. The hydrogen bonding parameters are given in Table 1. The existence of intermolecular hydrogen bonds between the molecules makes the interactions much stronger which would result in more stable and lower boiling points because the molecules prefer to be close vicinity with respect to each other. The stability of the dapsone molecule is also established by the high chemical hardness value (2.46a.u). Visualization of intermolecular interactions through the Hirshfeld surface analysis and obtaining the quantitative details of the contacts through the fingerprint analysis [28] are necessary to understand qualitatively and quantitatively the intermolecular interactions.

**Table 1:** Hydrogen bonding parameters (Åo)

D---H…A	D--H	H...A	D…A	D---H…A
N1–H9…O2	1.009	1.990	2.956	159.46
N1–H10…N1	1.009	2.777	3.269	163.31
N2–H12…C9	1.009	2.743	3.369	120.47
N2–H11…O1	1.009	2.423	3.623	145.29
C11–H7…C7	1.083	2.728	3.303	139.76

**Fig. 3:**
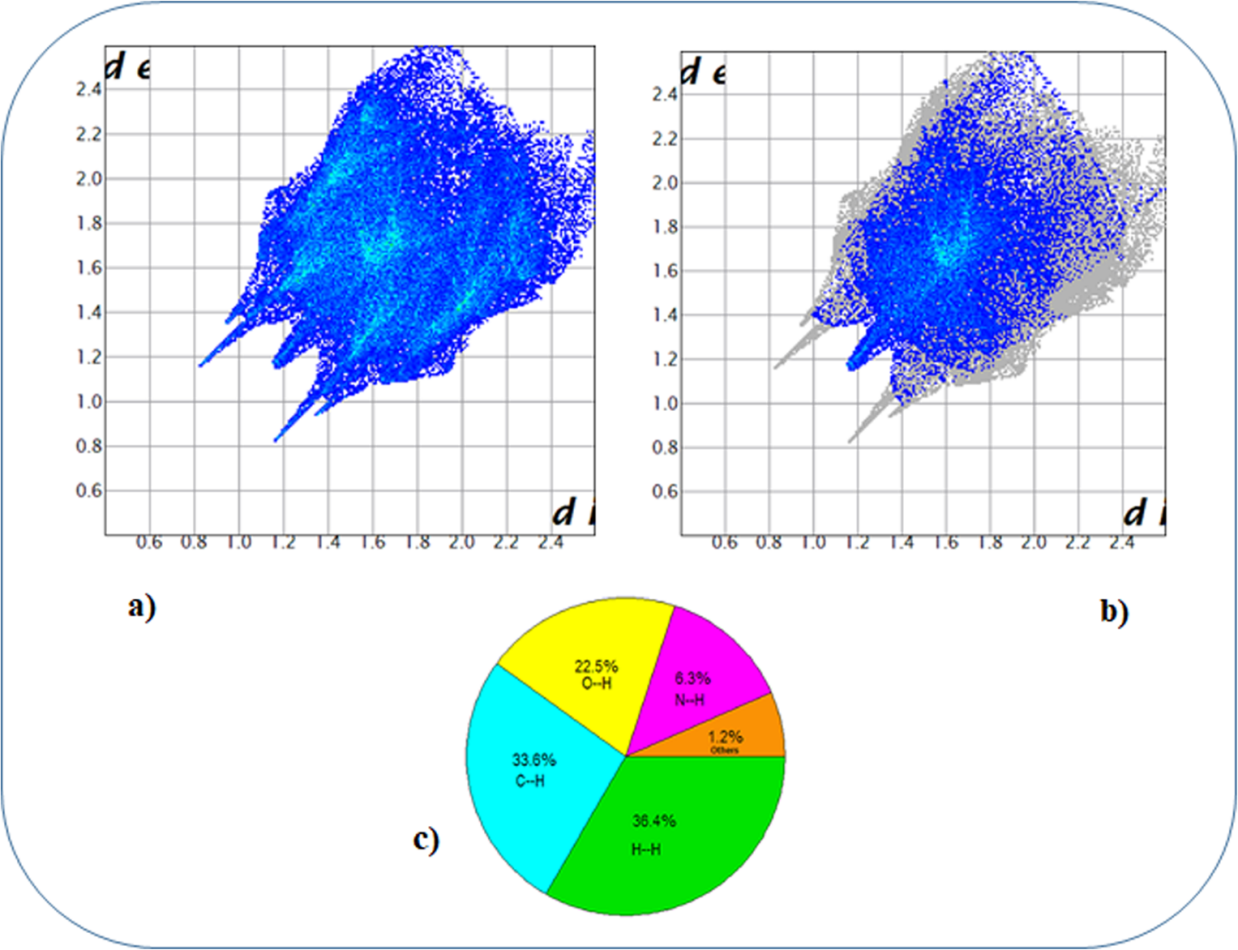
Fingerprint plot **a** full and **b** resolved into H…H contacts. **c** Percentage contribution of various intermolecular contacts in the molecule

The combination of *di* and *de* in the form of a 2D fingerprint plot is used to provide ample information regarding the percentage contribution of various intermolecular contacts present in the molecule. Figure 2b shows thick orange color circular patches which represent the strong N1–H9…O2, N1–H10…N1, and N2–H11…O1 hydrogen-bonding interactions which serve as an attestation for the strong intermolecular interactions. The fingerprint plot of the dapsone molecule (Fig. 3a) shows two sharp spikes at the bottom left and bottom right representing the donor and acceptor of N–H…O interaction. The distinct spike of the H…H type interaction is shown in Fig. 3b. Among the investigated interactions, H…H interaction has a major contribution (36.4%), while the C…H interaction occupies 33.6% of the area of the total Hirshfeld surface. The various intermolecular contacts and their percentage contributions are shown in Fig. 3c.

### Topological properties of electron density

**Fig. 4:**
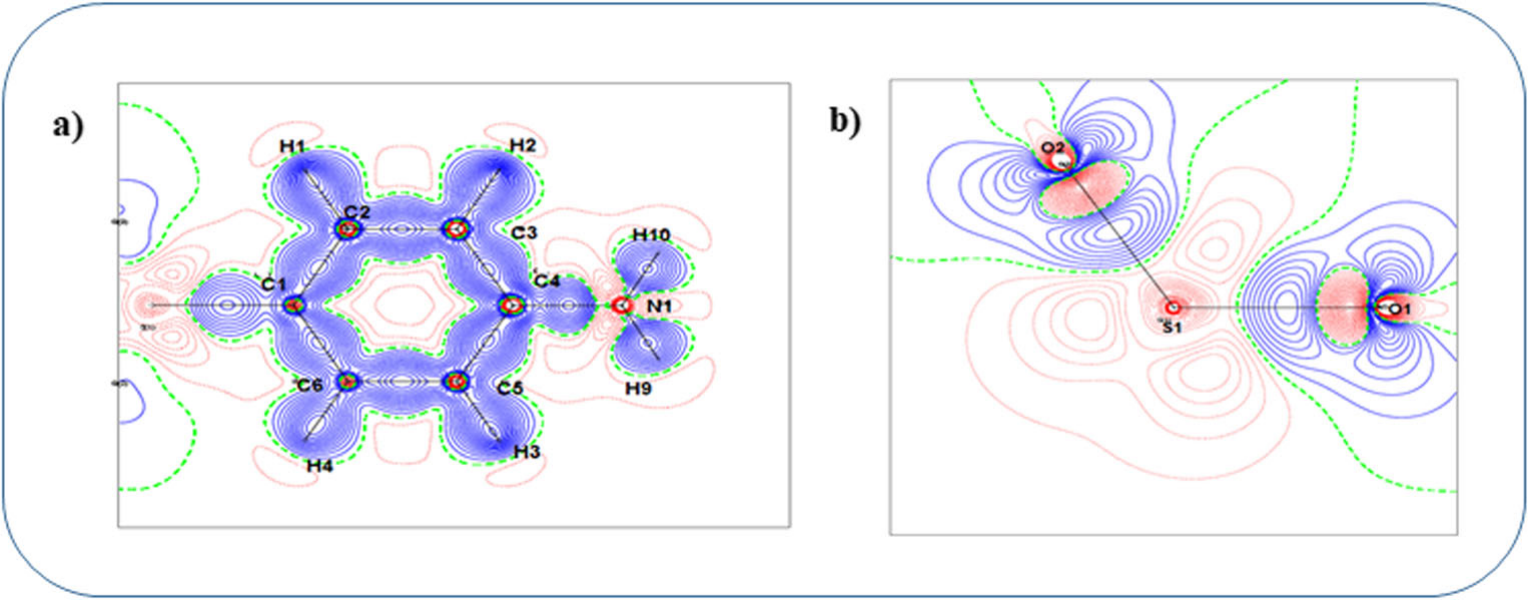
Static deformation density map of the **a** top aniline ring and **b** SO2 group of the dapsone molecule

Investigation on topological properties of electron density in the light of AIM theory allows us to look into the details of atomic, molecular, and chemical bonding. Figure 4a shows the static deformation density map of the top aniline ring where all the bonds exhibit covalent nature (Table 2). The closed-shell interactions between S–O atoms are clearly visible in Fig. 4b.

**Table 2:** Topological properties of the dapsone molecule

Bonds	*ρBCP* (e/Å3)	∇^2^*ρ* (e/Å5)	*ε*	*V*(r) (a.u.)	*G*(r) (a.u.)	*K*(r) (a.u.)	*L*(r) (a.u.)	DI
C1–C2	2.06	− 20.2	0.20	− 0.411	0.101	0.310	0.209	1.34
C2–C3	2.10	− 20.8	0.22	− 0.425	0.104	0.320	0.216	1.42
C3–C4	2.05	− 20.1	0.21	− 0.401	0.096	0.305	0.209	1.28
C4–C5	2.05	− 20.1	0.21	− 0.401	0.096	0.305	0.209	1.28
C5–C6	2.10	− 20.8	0.22	− 0.425	0.104	0.320	0.216	1.34
C1–C6	2.06	− 20.2	0.20	− 0.411	0.101	0.310	0.209	1.28
C7–C8	2.06	− 20.2	0.20	− 0.411	0.101	0.310	0.209	1.34
C8–C9	2.10	− 20.8	0.22	− 0.425	0.104	0.320	0.216	1.42
C9–C10	2.05	− 20.1	0.21	− 0.401	0.096	0.305	0.209	0.86
C10–C11	2.05	− 20.1	0.21	− 0.402	0.096	0.305	0.209	1.29
C11–C12	2.10	− 20.8	0.22	− 0.425	0.104	0.320	0.216	1.34
C7–C12	2.06	− 20.2	0.20	− 0.411	0.101	0.310	0.209	1.29
C1–S1	1.36	− 9.14	0.09	− 0.190	0.048	0.142	0.095	0.86
C7–S1	1.36	− 9.12	0.09	− 0.189	0.048	0.142	0.094	1.42
C4–N1	2.04	− 21.2	0.07	− 0.570	0.175	0.395	0.219	1.07
C10–N2	2.04	− 21.2	0.07	− 0.567	0.174	0.393	0.219	1.07
C2–H1	1.92	− 23.9	0.01	− 0.319	0.035	0.283	0.248	1.42
C3–H2	1.88	− 22.9	0.03	− 0.318	0.040	0.277	0.237	0.93
C5–H3	1.88	− 22.9	0.03	− 0.318	0.040	0.277	0.237	0.96
C6–H4	1.92	− 23.9	0.01	− 0.319	0.035	0.283	0.248	0.84
C8–H5	1.92	− 23.9	0.01	− 0.319	0.035	0.283	0.248	0.93
C9–H6	1.88	− 22.9	0.03	−0.318	0.040	0.277	0.237	0.96
C11–H7	1.88	− 22.9	0.03	− 0.318	0.040	0.277	0.237	0.96
C12–H8	1.92	− 23.9	0.01	− 0.319	0.035	0.283	0.248	0.93
S1–O1	1.93	20.3	0.01	− 0.899	0.555	0.344	− 0.210	1.19
S1–O2	1.93	20.3	0.01	− 0.899	0.555	0.344	− 0.210	1.19
N1–H9	2.30	− 38.5	0.06	− 0.513	0.057	0.456	0.399	0.84
N1–H10	2.30	− 38.5	0.06	− 0.513	0.057	0.456	0.399	0.96
N2–H11	2.30	− 38.5	0.06	− 0.512	0.057	0.456	0.399	0.84
N2–H12	2.30	− 38.5	0.06	− 0.512	0.057	0.456	0.399	0.84

**Fig. 5:**
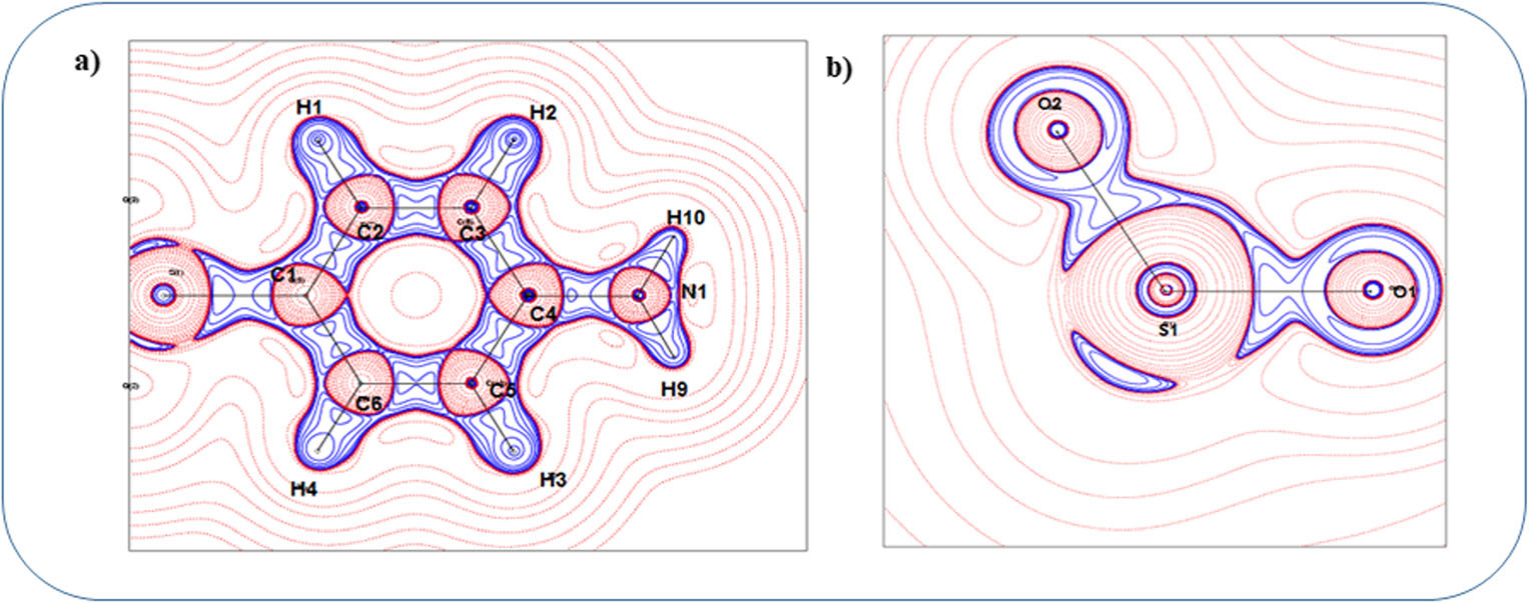
Laplacian of the electron density of **a** top aniline ring and **b** SO2 group of the dapsone molecule

**Fig. 6:**
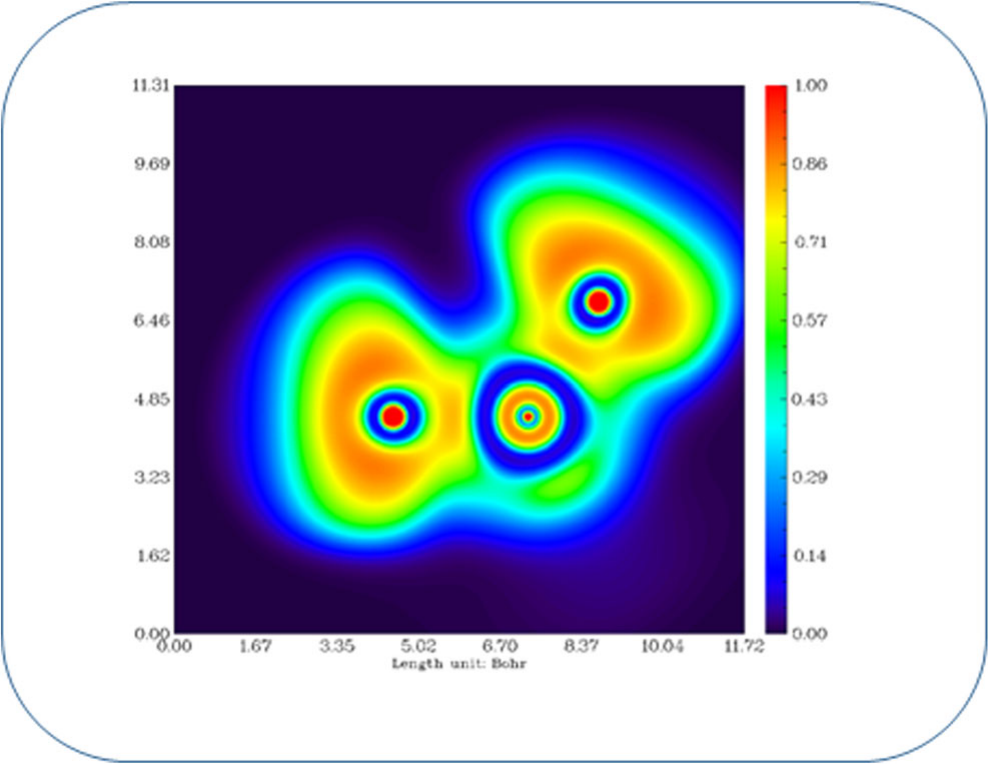
Visualization of electron localization function

**Fig. 7:**
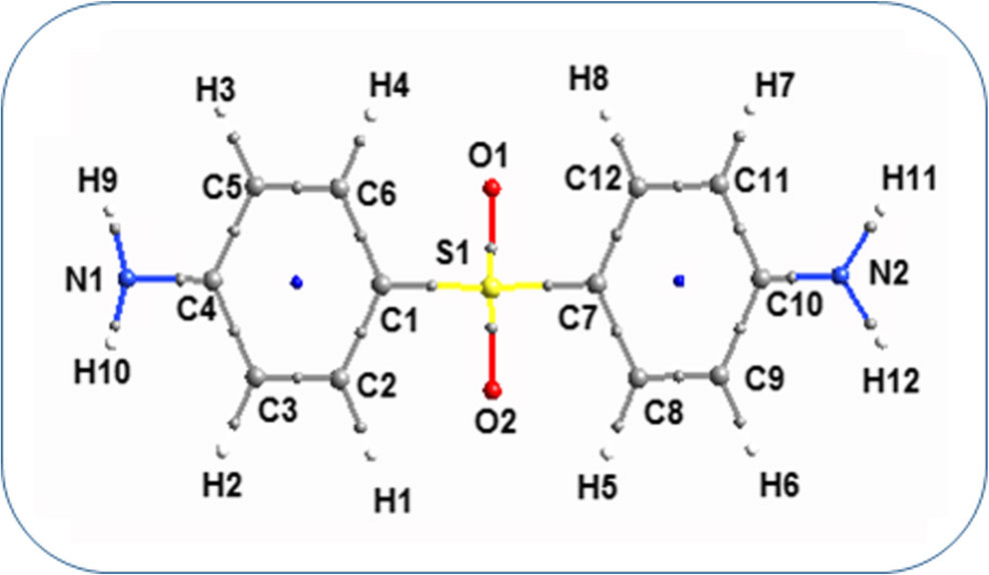
Molecular graph diagram with a bond (gray small circles) and ring (blue small circles) critical points of dapsone molecule

The range of electron density and Laplacian of C–C bonds in both the phenyl rings is 2.05e/Å3 to 2.105e/Å3 and − 20.1e/Å5 to − 20.8e/Å5. The C–S bonds exhibit very poor electron density as well as Laplacian values which shows that the bonds are weak. Both the C–N bonds show the same charge density and Laplacian values and are found to be 2.045e/Å3 and − 21.25e/Å5, respectively, and this might be stabilized with same charge environment. Similarly, the N–H and C–H bonds show good accumulation of charge density between the bonds (Fig. 5a). In addition to the electron density, the Laplacian and electron localization function also reveal the closed-shell interacting nature of the S–O bonds with the positive Laplacian value (20.35e/Å5) as well as through the plots of Figs. 5b and 6. The lone pairs of O atoms and the K, L, and M shells of S atom are also clearly visible in Fig. 6. The molecular graph with the bond as well as ring critical point has been shown in Fig. 7.

The very low ellipticity value (ca 0.01) for the bonds S–O and C–H portrays the near cylindrically symmetric nature. The ellipticity values for the C–C bonds are in the range 0.20–0.22, and the increased values show the π character of the C–C bonds.

Among the C–C bonds, the bonds C2–C3 and C8–C9 have the highest delocalization index value which shows not only the strength of the bonds but also denotes the occurrence of resonance/delocalization of the bonds in both the phenyl rings. Among the C–H bonds, the C2–H1 bond has the highest delocalization index value which is supported by the high charge density values (1.92e/Å3) as well as high Laplacian values (− 23.9e/Å5). Similarly, the C7–S1 bond exhibits a high value of the delocalization index (1.42) even though the charge density values of both the C–S bonds are the same. This might be because of the influence of the S1 atom that is higher in C7–S1 bond than the C1–S1 bond.

As the kinetic energy density dominates, the total energy density becomes positive which shows the instability of the bonds. Among all the bonds, the N–H bonds have high total energy density values which show the low stability of the bonds.

### Atomic charges and volume

The sulfur atom exhibits a very high positive Bader atomic charge (2.36e) as it is attached with the two most electronegative oxygen atoms (− 1.27e) on both sides. The electronegative N1 and N2 atoms exhibit negative charges (− 1.07e), and they are attached with the two positive H atoms each. The group charge of both the primary amine (−NH2) is negative − 0.23e), and they can donate electrons as donors. Both the C–N bonds are well polarized where the carbon atoms (C4, C10) possess positive charges (0.40e) and the nitrogen atoms (N1, N2) possess negative charges (− 1.07e). The most electronegative atoms O1 and O2 exhibit greater volume, and the values are found to be 111.18 Å3 and 111.19 Å3 respectively (Table 3).

**Table 3:** Bader charges and atomic volumes

Atoms	Q(e)	V(Å3)	Atoms	Q(e)	V(Å3)
S1	2.364	54.74	C11	− 0.011	72.05
O1	− 1.267	111.18	C12	0.014	69.36
O2	− 1.267	111.19	H1	0.064	35.25
N1	− 1.074	97.69	H2	0.016	37.58
N2	− 1.072	97.67	H3	0.016	37.59
C1	− 0.155	68.12	H4	0.064	35.26
C2	0.014	69.36	H5	0.064	35.23
C3	− 0.011	71.99	H6	0.016	37.62
C4	0.404	54.69	H7	0.016	37.62
C5	− 0.011	72.01	H8	0.064	35.22
C6	0.014	69.36	H9	0.374	23.84
C7	− 0.155	68.09	H10	0.374	23.83
C8	0.014	69.33	H11	0.373	23.86
C9	− 0.011	72.02	H12	0.373	23.86
C10	0.402	54.68			

### Physiochemical properties: PASS analysis

Some of the molecular properties like membrane permeability and bioavailability are straightly connected with some molecular descriptors such as log P (partition coefficient), molecular weight (MW), hydrogen bond acceptors, and donors count in a molecule. Lipinski rule of five [29] which is also used as a filter for drug-like properties says that most molecules with good membrane permeability have log P ≤ 5, molecular weight ≤ 500, number of hydrogen bond donors ≤ 5, and number of hydrogen bond acceptors ≤ 5. Lipinski rule of 5 parameters has been listed in Table 4. The compound satisfies the above conditions and proves itself as a potent drug and physiologically active.

**Table 4:** The physiochemical properties of the dapsone molecule

Lipinski rule of 5 parameters	
1. mi Log P	− 0.70
2. TPSA	86.19
3. MW	260.4
4. HBA	4
5. HBD	4
6. No. of violations	0
7. No. of rotatable bonds	2

### Electrostatic potential

**Fig. 8:**
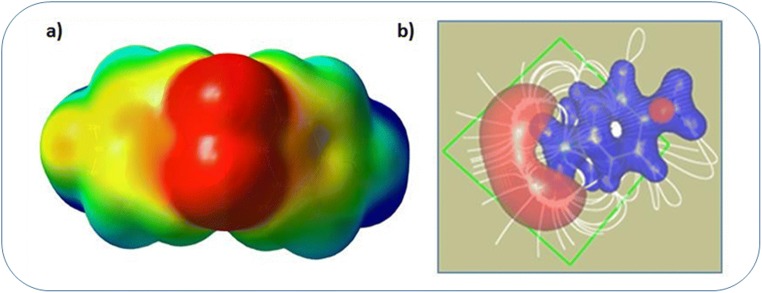
**a** The isosurface representation of the molecular electrostatic potential of the dapsone molecule. **b** Lock and key type structure with gridlines of the dapsone molecule

The electrostatic potential is primarily used to trace out the electrostatic positive as well as electrostatic negative regions where the ligand-receptor interactions possibly occur [30]. An asymmetric potential is obtained for the dapsone molecule, and the mapped electrostatic potential surfaces are shown in Fig. 8a where the negative region of the molecule seems to concentrate mainly on O2–S1–O1 (Fig. 8b), and it envelopes mainly on the oxygen atoms O1 and O2 as their respective negative charges are − 1.27e and the average bond length (S1–O1, S1–O2) is 1.47 Å. The color gradient from blue to red shows the positive and negative regions of ESP which pronounces the electrophilic and nucleophilic regions of the molecule. The enveloping of negative nucleophilic to the positive electrophilic seems to enact the lock and key structure as observed in protein-ligand interaction [31]. Figure 8b shows the negative region (red) surrounding O1 and O2, whereas the positive regions (blue) of the molecule show the complete circuits of electric field gridlines, thus distinguishing electrophilic and nucleophilic regions. The grid lines showing the electric field are drawn for the bottom phenyl ring (Fig. 8b) at the isosurface value level 0.24 (blue) to − 0.038 (red) which show the origin of the field grid lines centered at the negative envelope surrounding the atoms O1 and O2 and end up with the positive hydrogen ions.

As shown in the lock and key shape of the molecular electrostatic potential of dapsone, the negative potential which is in the vicinity of O1 and O2 atoms is susceptible to severe electrophilic attack. When dapsone is taken as an antibacterial, the nucleophilic/electrophilic region of dapsone interacts with the electrophilic/nucleophilic region of the dihydrofolic acid, and the electrophilicity of the new molecule gets equalized by the electronic charge transfer between the ligand and the receptor [32]. In this manner, dapsone inhibits the growth of a bacterial cell by discontinuing dihydrofolic acid synthesis.

## Molecular interaction of dihydropteroate synthase (DHPS) and dapsone

### Homology modeling

The crystal structure of DHPS is not available in protein database. Therefore, the proteomic sequence of DHPS (Uniprot ID: P0C0X1) of *Mycobacterium leprae* was obtained from Uniprot database. The homology modeling of extracted protein sequence was given as an input on the automated SWISS-MODEL server [33]. Based on the integrated deep view method in the server, the complex crystal structure of DHPS of *Mycobacterium tuberculosis* was selected as a template (PDB ID: 1EYE). Finally, the refined structure was used for further studies.

### Ligand preparation

Two-dimensional structure of dapsone (CID: 2955) was obtained from PubChem database. The .sdf file converted into .pdb by using Open Babel tool [34].

### Molecular docking

The molecular docking was performed by using Molecular Graphics Laboratory AutoDock (4.2) tools. The preparation of receptor and ligand, grid, and docking calculation was described in the previous studies [35].

### Molecular interaction of DHPS and dapsone

**Fig. 9:**
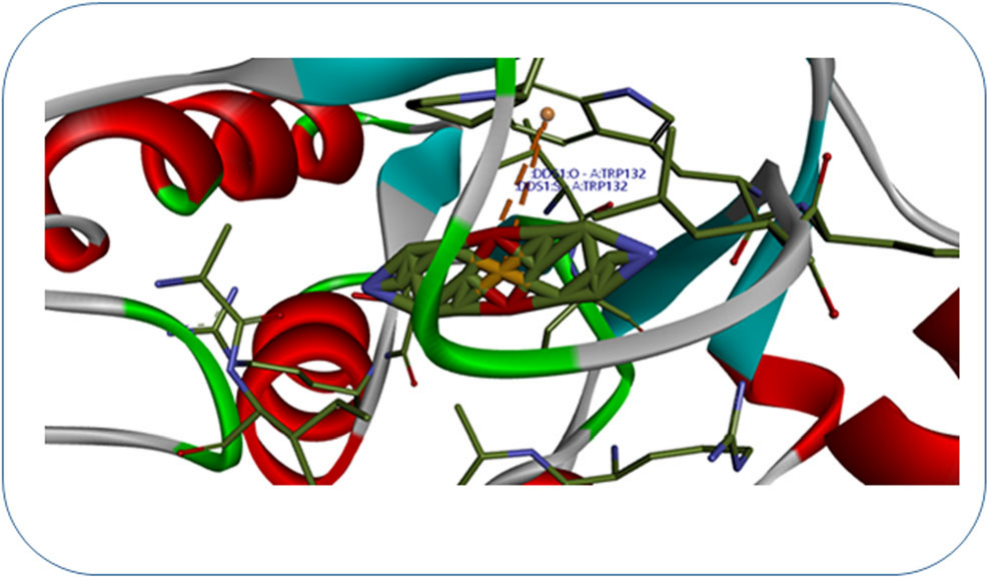
Non-covalent π-cation interactions of dihydropteroate synthase (DHPS) and dapsone. The interacting residues were denoted in blue in color

Molecular docking calculation of dapsone with target enzyme dihydropteroate synthase (DHPS) generated six clusters of conformers using a root mean square difference (RMSD) tolerance of 2.0°A. The two non-covalent π-cation interactions estimated on the amino acid residues TRP 132 of DHPS with respective oxygen and sulfur atoms of dapsone (Fig. 9). The results suggested that dapsone have played an important role in the dihydrofolic acid synthesis.

## Conclusions

We have determined the theoretical charge density distribution for the gas phase of dapsone on the basis of the AIM theory. The identification of close contacts and distinguishing them from each other has been accomplished through the Hirshfeld surface analysis and the fingerprint plots. The topological analysis reveals the strength and chemical bonding details of all the covalent bonds in the molecule. The subshells of S atom and lone pairs of O and N atoms are visible in the ELF plots, and the ELF clearly shows the closed-shell interaction of the S–O bonds. In the ESP map, a high electronegative region is found in the vicinity of the O1 and O2 atoms, and this is the active site for electrophilic attack. This theoretical charge density study clearly provides the fine details of structural information, intermolecular interaction, and charge density distribution which are the necessary parameters to interpret the drug-receptor interactions between the dapsone molecule and enzyme of dihydropteroate synthase (DHPS).
